# Cerebral‐Cerebellar Cortical Activity and Connectivity Underlying Sensory Trick in Cervical Dystonia

**DOI:** 10.1002/acn3.52177

**Published:** 2024-08-16

**Authors:** Nai‐Qing Cai, Wu‐Xiang Shi, Ru‐Kai Chen, Bo‐Li Chen, Yu‐Rong Li, Ning Wang

**Affiliations:** ^1^ Department of Neurology, the First Affiliated Hospital Fujian Medical University Fuzhou 350005 Fujian China; ^2^ Department of Neurology, National Regional Medical Center, Binhai Campus of the First Affiliated Hospital Fujian Medical University Fuzhou 350212 Fujian China; ^3^ Fujian Key Laboratory of Molecular Neurology Fujian Medical University Fuzhou 350005 Fujian China; ^4^ Department of Fujian Provincial Key Lab. of Medical Instrument and Pharmaceutical Technology Fuzhou University Fuzhou 350108 Fujian China; ^5^ College of Electrical Engineering and Automation Fuzhou University Fuzhou 350108 Fujian China

## Abstract

**Objective:**

The objective of this study was to investigate the activity and connectivity of cerebral and cerebellar cortices underlying the sensory trick (ST) effects in patients with cervical dystonia (CD), using electroencephalography (EEG).

**Methods:**

We recruited 15 CD patients who exhibited clinically effective ST and 15 healthy controls (HCs) who mimicked the ST maneuver. EEG signals and multiple‐channel electromyography (EMG) were recorded simultaneously during resting and acting stages. EEG source analysis and functional connectivity were performed. To account for the effects of sensory processing, we calculated relative power changes as the difference in power spectral density between resting and the maneuver execution.

**Results:**

ST induced a decrease in low gamma (30–50 Hz) spectral power in the primary sensory and cerebellar cortices, which remained lower than in HCs during the maintenance period. Compared with HCs, patients exhibited consistently strengthened connectivity within the sensorimotor network during the maintenance period, particularly in the primary sensory‐sensorimotor cerebellum connection.

**Interpretation:**

The application of ST resulted in altered cortical excitability and functional connectivity regulated by gamma oscillation in CD patients, suggesting that this effect cannot be solely attributed to motor components. The cerebellum may play important roles in mediating the ST effects.

## Introduction

Sensory trick (ST) is a specific clinical sign in dystonia, involving voluntary movements to alleviate dystonic posturing.^1^ It has been reported to be effective in over 80% of patients with cervical dystonia (CD).^2^ The pathophysiological mechanism of CD has been proposed to involve a sensorimotor integration disorder with alterations in multiple brain regions within the sensorimotor circuit.^3^, ^4^, ^5^ However, the role of ST in the pathophysiology of CD remains poorly understood.

Previous studies utilizing various techniques such as transcranial magnetic stimulation, deep brain stimulation (DBS), functional magnetic resonance imaging (fMRI), positron emission tomography, and electrophysiological tests have indicated that ST can modulate sensorimotor integration by inducing changes in activity within sensorimotor cortices, including the primary motor cortex (M1), primary sensory cortex (S1), parietal cortex (including the supplementary motor area, SMA), and globus pallidus.^6^, ^7^, ^8^, ^9^, ^10^ Eelectroencephalography (EEG), known for its high temporal resolution, is a preferred tool for investigating cortical activity and may offer valuable insights into the ST effects. Recent work by Sang et al.^11^ assessed corticocortical coherence in different brain regions between CD patients and healthy volunteers, revealing that ST could enhance cortical function, thereby reducing corticomuscular connectivity and dystonia. Similarly, Nicoletta et al. compared ST‐related EEG spectral changes among CD patients and healthy controls (HCs), suggesting that both motor and sensory areas contribute to the ST effects.^12^ However, these studies only focused on channel‐level EEG signals, and the ST‐induced changes in the source level remain unclear due to the volume conduction effect.

Additionally, the cerebellum plays a crucial role in CD pathophysiology and is interconnected with the basal ganglia through the cerebellar‐basal ganglia‐thalamo‐cortical loop.^13^, ^14^ In a structural MRI study on CD, an increase in gray matter was noted in the anterior lobe of the cerebellum, whereas atrophy was concentrated in the posterior lobe.^15^ As per the topographic organization of the human cerebellum, lesions involving the anterior lobe and parts of lobule VI (termed the “sensorimotor cerebellum”) may disrupt motor systems, while lesions affecting lobules VI and VII (termed the “cognitive cerebellum”) are associated with cognitive impairments.^16^, ^17^ We postulated that the sensorimotor cerebellum and cognitive cerebellum are crucial functional areas involved in CD pathophysiology, inevitably associated with the ST mechanism as well, given the cerebellum's involvement in ST effects.^18^ However, it remains unclear whether and how the different cerebellar regions participate in the ST effects.

Based on the aforementioned evidences, we hypothesized that S1, M1, SMA, sensorimotor cerebellum, and cognitive cerebellum would be the most important areas involved in the ST procedure. We anticipated significant changes in activity in these regions of interest (ROIs) induced by employing ST. To deepen our understanding of the ST mechanism, we collected EEG and synchronized cervical electromyography (EMG) data from CD and healthy individuals throughout the ST/ST‐mimicking process and accurately identified the initiation of the action using EMG signals. Following EEG source localization on these ROIs, source activation and cortical functional connectivity were analyzed. This study aims to provide further evidence for potential therapeutic targets for patients with dystonia.

## Methods

### Participants

Twenty‐two isolated CD patients who had experienced effective ST and 15 HCs participated in this study. All participants were evaluated at the outpatient clinic of the First Affiliated Hospital of Fujian Medical University. Informed consent was obtained from each participant, adhering to the principles outlined in the Declaration of Helsinki. The study protocol received approval from the institutional local ethics committee (MRCTA, ECFAH of FMU [2021]163).

The disease severity was assessed using the Toronto Western Spasmodic Torticollis Rating Scale (TWSTRS).^19^ Patients were considered eligible for inclusion if they had demonstrated beneficial effects of ST. A beneficial effect was defined as any partial or complete correction of the head position by touching one side of the head or face when clinical observation confirmed the presence of ST improvement rates greater than 10%. The ST improvement rate was calculated as the decrease in scores pre‐ and post‐ST divided by the scores pre‐ST. These scores comprised the sum of parts of “Part I. Torticollis Severity Scale” from TWSTRS, including “A. Maximal Excursion,” “D. Shoulder Elevation/Anterior Displacement,” “E. Range of Motion,” and “F. Time.”

Exclusion criteria included (1) Comorbidity with neck tremor or jerk, (2) botulinum toxin injection within 3 months preceding the testing session, (3) presence of other neurological diseases, (4) intake of drugs interfering with brain activity, (5) presence of comorbidities significantly influencing ST maneuver execution (e.g., history of orthopedic ailments, cardiopulmonary diseases, and mental disorders), and (6) DBS.

### EEG and EMG recording

We conducted continuous EEG data recordings using a Neuroscan Quick‐cap (SynAmps2, Neuroscan Inc, Herndon, USA) equipped with 64 channels distributed according to the 10–20 international system. The reference electrodes were positioned on the subject's ears, while the ground electrode was affixed to the prefrontal lobe. EEG data were sampled at a rate of 1000 Hz using the SynAmp2 8050 amplifier, with electrode impedance maintained at ≤10 kΩ.

Surface EMG signals from the affected limb were captured using the DELSYS Trigno™ wireless EMG system, operating at a sample rate of 2000 Hz. Muscles typically monitored included the sternocleidomastoid, trapezius, splenius capitis, levator scapulae, and biceps brachii muscles, which are bilateral in the body. EMG electrodes were positioned on the skin surface over the muscle bellies of selected muscles. Continuous EMG–EEG co‐registration was carried out throughout the experimental procedures.

### Experimental protocol

**Figure 1:**
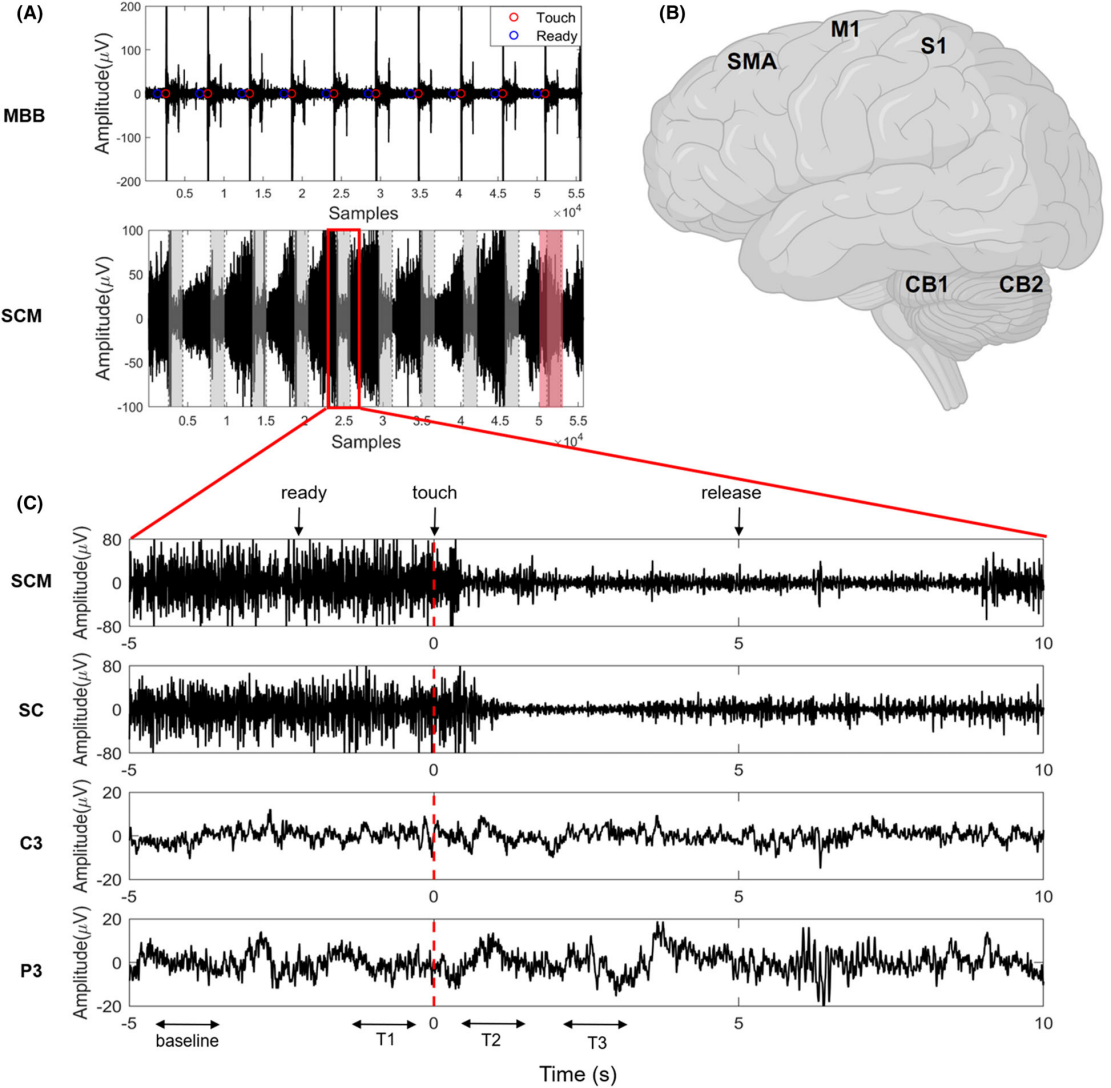
Experimental protocol and EEG–EMG signal processing. (A) The given prompts of “Touch” (red circles) were followed by the onset of the MBB contraction, with the EMG signal of the SCM significantly decreasing. The experiment comprised 10 trials. Trials with EMG improvement rates <30% were removed (red shaded area). (B) Five ROIs were confirmed based on source localization. (C) One single trial was zoomed in. Synchronized EEG–EMG signals were continuously recorded. We divided each trial into four time periods based on the EMG signal onset of the MBB (the red broken line), including resting period (baseline), the preparation period (T1), the action period (T2), and the maintenance period (T3). CB1, sensorimotor cerebellum; CB2, cognitive cerebellum; M1, primary motor cortex; MBB, musculus biceps brachii; S1, primary sensory cortex; SC, splenius capitis; SCM, sternocleidomastoid; SMA, supplementary motor area.

Participants were situated in a tranquil, distraction‐free setting with a computer monitor positioned in front of them. Patients were instructed to touch the side of their head or face using the ipsilateral hand that triggered the best ST effect, and then release the touch to return to the rest position. Conversely, in order to avoid additional activity changes in sensorimotor cortices caused by voluntary head movements, HCs were directed to mimic ST with their heads in the neutral position, by using their right hand to touch their right faces and then return to the rest position. All participants executed the tasks in response to prompts (“rest,” “ready,” “touch,” and “release”) provided by the computer (Fig. 1). The order of the remaining conditions was counterbalanced across participants. Each condition was thoroughly explained, practiced if necessary, and then recorded. Any interruptions necessitated restarting the experiment.

The experiment comprised 10 trials with no intertrial interval, during which synchronized EEG–EMG signals were continuously recorded.

### Signal processing

EEG/EMG signal processing using custom scripts developed in MATLAB. Initially, both EEG and EMG data were down sampled to 256 Hz. We preprocessed the EEG and EMG signals using a Butterworth bandpass filter. Specifically, the EEG signal underwent filtering within the 1–80 Hz frequency range, while the EMG signal was filtered across the 20–200 Hz spectrum. Additionally, a notch filter (50 Hz) was applied to mitigate line noise. This filtering approach was based on the observed spectral characteristics within the EEG and EMG signals.

Given the susceptibility of scalp surface EEG signals to contamination by artifacts, we employed canonical correlation analysis to remove muscle artifacts from the EEG signal. Independent component analysis was utilized to eliminate ocular artifacts. To unify the EEG of CD patients performing ST with their left hand and those performing the ST with their right hand, this study adopted a signal flipping strategy. Specifically, flip the EEG signals generated by the left‐hand performing ST horizontally along the medial interhemispheric fissure line to achieve consistency with the EEG signals generated by the right‐hand performing ST.

Furthermore, we extracted the signal envelope of the subjects' biceps brachii through rectification and low‐pass filtering. Subsequently, the signal underwent a Teager–Kaiser Energy Operator (TKEO) transformation to reduce sensitivity to noise. Finally, we identified the initiation and termination points of muscle activation by applying a threshold set at 1% of the signal's peak value. The calculation formula for TKEO is as follows:

(1) $$\psi \left(x\left(n\right)\right)=x^{2}\left(n\right)-x\left(n+1\right)\cdot x\left(n-1\right)$$

We synchronized the EEG signals with the EMG signals by identifying the activation onset of the biceps brachii muscle when the subject lifts their hand. This method effectively addresses the issue of inconsistent reaction times among subjects in executing movements following cues. We divided each trial into four time periods for data analysis based on the activation onset of the bicep brachii muscle EMG (0 s). These periods were defined as follows: baseline for the resting period (−5 to −4 s), T1 for the preparation period (−1.5 to −0.5 s), T2 for the action period (0 to 1 s), and T3 for the maintenance period (3 to 4 s). The detailed methods of calculation and time tags are illustrated in Figure 1.

During the data collection phase, we observed that the effectiveness of ST could be affected in some patients wearing electrode caps. Additionally, factors such as anxiety may also contribute to the failure of the ST in certain cases. Previous research has indicated a notable reduction in the activation levels of muscles impacted in CD patients during the execution of ST.^12^ To explore the mechanism of action of ST more accurately, we calculated the proportion of decreased activation amplitude of affected muscles before and after executing ST. We screened all trials using a threshold of 30%. Trials with EMG improvement rates less than 30% indicate that the effect of ST on the subjects is not significant, and therefore, these trials will be removed. Furthermore, patients with fewer than three effective trials after screening will be directly excluded. The screening process is illustrated in Figure 1, where the red shaded area indicates that this trial was not qualified.

### Time–frequency analysis

**Figure 2:**
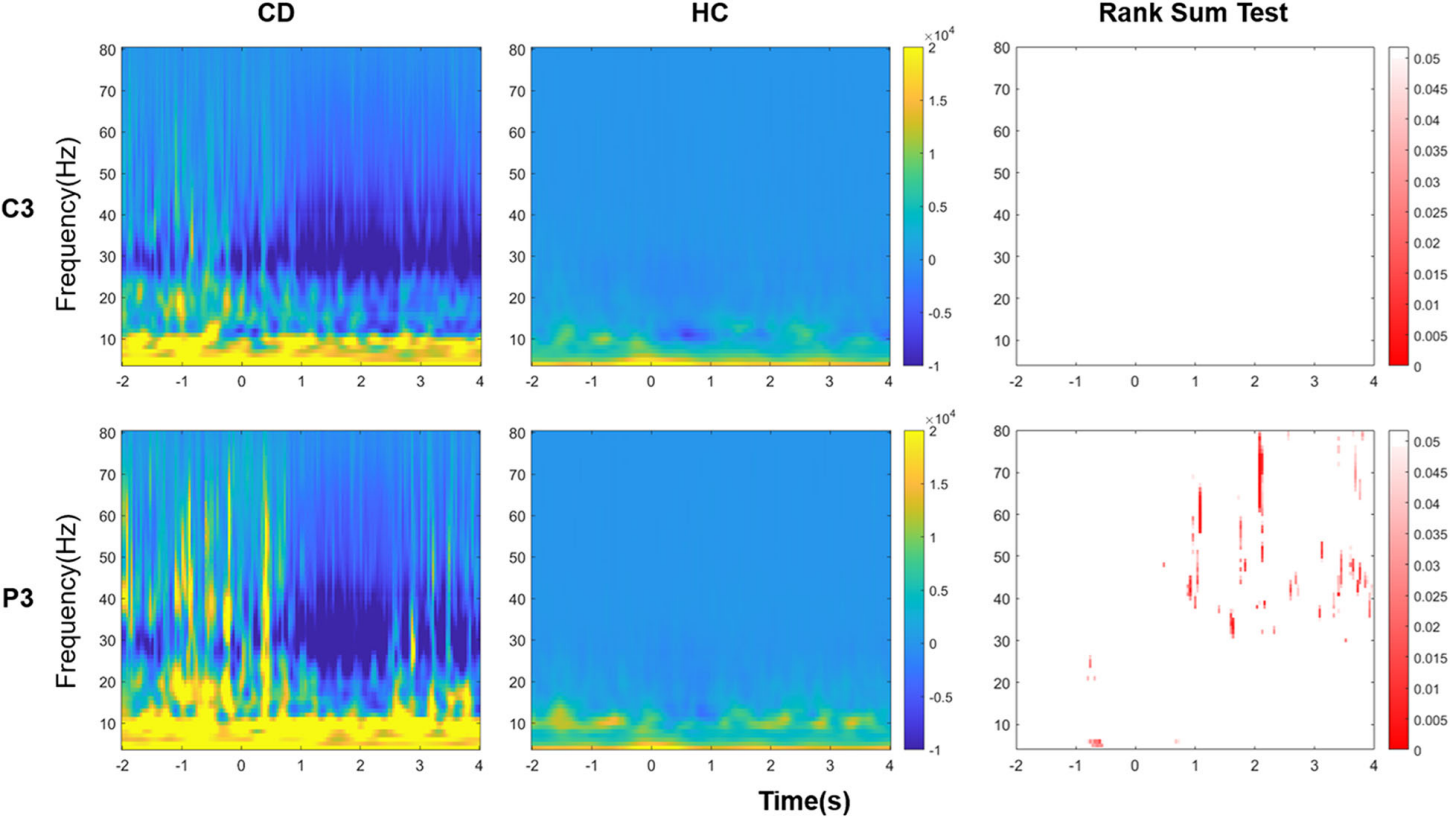
Time–frequency analysis and the rank sum test between CD patients and HCs during ST/ST mimicking. Point 0 is marked based on the EMG signal onset of the musculus biceps brachii. The time–frequency analysis of CD patients (left column) showed a significant decreasing power over low gamma frequency band (30–50 Hz) after performing ST. In contrast, the time–frequency analysis of HCs (middle column) showed a decreasing power around 10 Hz. The rank sum test after FDR correction (right column) showed significant differences in the low gamma frequency band of the channel P3 between two groups (non‐significant parts: white, significant parts: red). The channel P3 represents sensory cortex. The channel C3 represents motor cortex. CD, cervical dystonia; HC, healthy control.

To investigate the specific cortical oscillatory activity of CD patients during ST, it is imperative to initially ascertain the frequency range of these oscillations. We employed time–frequency (TF) decomposition to meticulously examine the activity within the channel C3 and P3 during the ST task, which are important areas for motor and sensation function. The TF analyses were conducted using Morlet wavelets. The results of the TF analysis are depicted in Figure 2, illustrating the dynamic changes in signal characteristics over time and frequency domains. The figure reveals a pronounced event‐related desynchronization (ERD) around 30 Hz following the implementation of ST in patients, which is not significantly observed in healthy subjects.

To gain a clearer understanding of the disparities in TF power spectra between patients and healthy subjects, rank sum tests were conducted on the outcomes of both groups, and the *p* values were corrected using the false discovery rate (FDR) method. The results of the rank sum test are presented on the far right of Figure 2. In the figure, significant differences (*p* ≤ 0.05) are marked in red, while non‐significant differences (*p* > 0.05) are shown in white. The findings indicate no significant difference in the motor regions (C3) of the brain, while there is a significant difference in the sensory regions (P3) of the brain in the gamma frequency band, particularly in the low gamma frequency band (30–50 Hz), between patients and healthy subjects during the execution of ST.

### EEG source localization

However, the spatial resolution of scalp surface EEG signals is inherently low due to the volume conduction effect, which means that multiple brain source activities are simultaneously captured by a scalp electrode, making it difficult to find the brain regions that are truly activated during the execution of ST. Therefore, to attain a deeper understanding of the neural mechanisms of ST, we traced the EEG signals recorded on the scalp surface back to the interior of the brain for analysis.

For this purpose, we employed dynamic imaging of coherent sources (DICS), a beamforming technique in the frequency‐domain. DICS is specifically designed for identifying the cortical sources and synchronization within a predefined frequency band (30–50 Hz). This method has been successfully utilized in reconstructing the sources of frequency‐specific activity in various EEG and MEG studies.^20^

For source localization, we employed the DICS beamformer using the implemented forward model of Fieldtrip.^21^ The TF window chosen for source localization was determined based on the data analyzed at the electrode level (as described above). After aligning the EEG electrodes with the forward model, we computed the lead field matrix by dividing the brain volume of the forward model into grids with a 5 mm resolution. Subsequently, the lead field matrix was calculated for each grid point, with the regularization parameter set to 5%. Additionally, the weights of the spatial filter were standardized to address depth bias. To mitigate the influence of brain background and focus more on analyzing the differences caused by ST, we performed baseline correction on the power of sources, utilizing relative power $S_{T}^{\prime }$ instead of absolute power $S_{T}$, as shown below:

(2) $$S_{T}^{\prime }=\frac{\left(S_{T}-S_{\text{baseline}}\right)}{S_{\text{baseline}}}$$

where $S_{\text{baseline}}$ represents the source power during the resting period.

Subsequently, we utilized the AAL brain template to segment all voxels in the entire brain into 116 distinct regions. From these, we extracted five ROIs for our analysis: SMA, the contralateral S1 and M1, the ipsilateral sensorimotor cerebellum and cognitive cerebellum to the hand implementing ST. This selection was guided by the hypothesis that these specific areas play crucial roles in the neural mechanisms and processes that are under investigation in our study, thereby focusing our analysis on the regions that are crucial for understanding the phenomena of interest.

Furthermore, we conducted coupling analysis on the selected regions of interest by examining the imaginary parts of coherence (iCOH), a method that enables the investigation of functional connectivity between these areas, focusing on the phase relationship of oscillatory activity while mitigating volume conduction effects.

### Statistical analysis

We analyzed both the source power and functional connectivity of the ROI using SPSS Statistics 26. The Shapiro–Wilk test results indicate that the data for each group adhere to a normal distribution. To evaluate the effect of ST, we employed mixed‐design ANOVAs to test the effect of the factors time (T1, T2, and T3), region (S1, SMA, M1, sensorimotor cerebellum, and cognitive cerebellum), and group (CD patients and HCs) on source power and functional connectivity. Time and region were considered within‐subject factors, while group was regarded as the between‐subjects factor. We adjusted the *p* value using the Greenhouse–Geisser Epsilon method in case of sphericity violation (Mauchly's test). All statistical results were adjusted using the FDR correction method.

## Results

### Study population

We finally included data from 15 out of 22 CD participants and 15 HCs in the analysis. Data from seven patients with EMG improvement rate < 30% were excluded. Participants' characteristics are presented in Table 1.

**Table 1:** Clinical characteristics of participants.

Characteristics	CD (*n* = 15)	HCs (*n* = 15)	*p* value
Age, years, mean (SD)	43.9 ± 12.2	27.4 ± 5.9	<0.001^a^
Sex, M/F	6/9	8/7	0.715^b^
Disease duration, years, median (range)	1.0 (0.17–17.0)	–	–
Main type of CD			
Laterocollis, *n* (%)	1 (7%)	–	–
Rotation, *n* (%)	12 (80%)	–	–
Lateral Shift, *n* (%)	2 (13%)	–	–
Type of ST			
Touching face with right hand, *n* (%)	8 (53%)	–	–
Touching face with left hand, *n* (%)	7 (47%)	–	–
TWSTRS I score, mean(SD)	12.3 ± 2.4	–	–
TWSTRS II score, mean(SD)	10.6 ± 5.5	–	–
TWSTRS III score, mean (SD)	3.9 ± 5.5	–	–
ST improvement rate, %, median(range)	87.5 (25–100)	–	–
EMG improvement rate, %, median(range)	49.3 (34.5–78.9)	–	–

CD, cervical dystonia; EMG, electromyography; ST, sensory trick; TWSTRS, Toronto Western Spasmodic Torticollis Rating Scale.

^a^ Independent samples *t*‐test.

^b^ Fisher exact test.

### EEG source analysis

#### ST Effects on Cortical Activation

**Figure 3:**
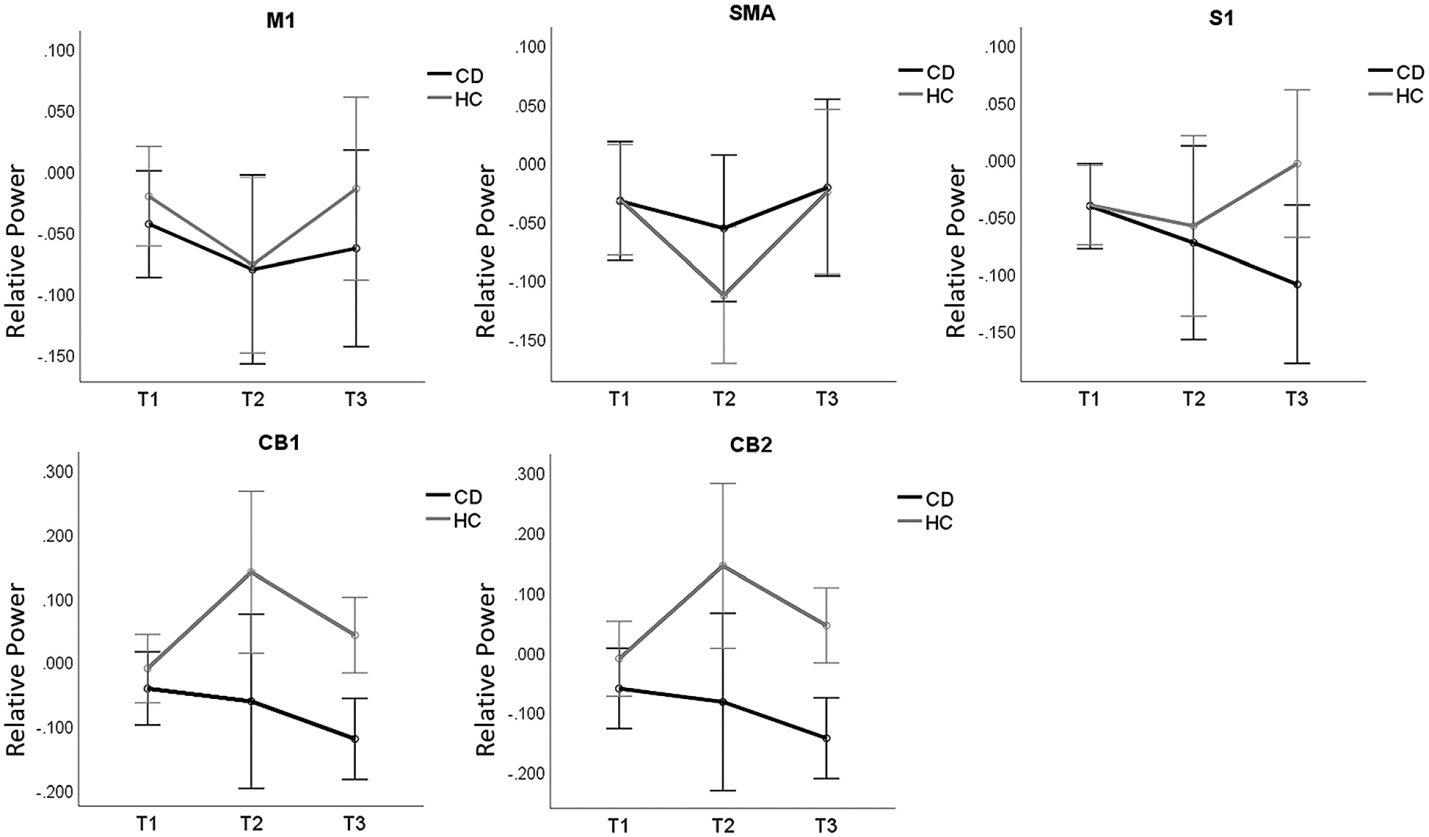
Source power of CD patients and HCs on ROIs. During the application of ST, the power of CD patients decreased significantly over S1, CB1 and CB2, remaining lower than that of HCs during the maintenance period. In contrast, the HC group, when mimicking ST, exhibited changes in spectral power over these areas, returning close to levels observed during the preparation period. The gray lines represent HCs. The black lines represent CD patients. CB1, sensorimotor cerebellum; CB2, cognitive cerebellum; CD, cervical dystonia; HC, healthy control; M1, primary motor cortex; S1, primary sensory cortex; SMA, supplementary motor area.

Mixed‐design ANOVAs on source power revealed a significant time* region* group interaction (*F* = 3.738, *p* = 0.030), as illustrated in Table S1 and Figure 3. We sought to delve into the specific distinctions in activation of various brain regions between CD patients and HCs in executing ST by examining the interaction between regions * group across different time levels.

So, we controlled the level of time and analyzed the interactions between regions and groups. Initially, no significant difference in source power between CD patients and HCs in various regions was observed at T1. However, a significant interaction effect (*F* = 6.554, *p* = 0.010) between CD patients and HCs in the group * region emerged at T2. This outcome suggests notable disparities in sensory processing and motor execution between CD patients and HCs. Post hoc tests with FDR correction revealed that in the cerebellum, the relative power of EEG source was notably lower (*F* = 4.275, *p* = 0.048) in CD patients compared with HCs.

Subsequently, a significant group * region interaction effect (*F* = 9.836, *p* < 0.001) was also observed at T3. Post hoc tests with FDR correction demonstrated that the relative power of EEG source in the central posterior gyrus, cerebellar cognition, and cerebellar sensorimotor regions was significantly lower (*p* = 0.046, *p* < 0.001, *p* < 0.001, respectively) in CD patients compared with HCs. Additionally, at T3, the source power levels in HC have roughly reverted to the baseline state observed before the execution of ST, while in patients, the source power in S1, sensorimotor cerebellum, and cognitive cerebellum has significantly decreased (*p* = 0.048, *p* = 0.020, *p* = 0.031, respectively) compared with the baseline state. Power reduction, known as the ERD phenomenon, signifies increased cortical activity in regions associated with the task, reflecting the recruitment of neural resources essential for processing, and responding to the task. These findings indicate significant activation in the S1, sensorimotor cerebellum, and cognitive cerebellum of CD patients during T3, contrasting with the absence of this phenomenon in HCs.

#### ST Effects on Functional Connectivity

**Figure 4:**
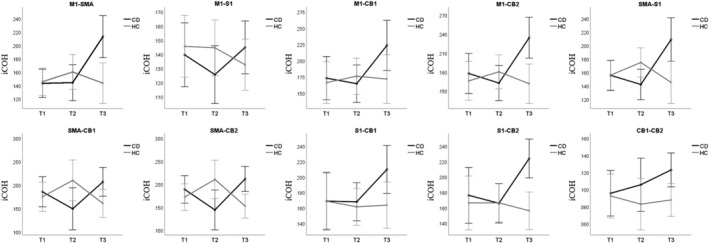
Functional connectivity of CD patients and HCs between ROIs. Compared with HCs, CD patients exhibited consistent strengthened connectivity within the sensorimotor network during the maintenance period, particularly in the S1‐CB1 connection. In CD patients, a temporary decrease in functional connectivity of SMA‐CB1 and SMA‐S1, followed increases in connectivity between overall ROIs except M1‐S1 and M1‐CB1. The gray lines represent HCs. The black lines represent CD patients. CB1, sensorimotor cerebellum; CB2, cognitive cerebellum; CD, cervical dystonia; HC, healthy control; M1, primary motor cortex; S1, primary sensory cortex; SMA, supplementary motor area.

Mixed‐design ANOVAs on functional connectivity revealed a significant time * group interaction (*F* = 7.761, *p* < 0.001), as detailed in Table S2 and illustrated in Figure 4. This interaction can be attributed to differential changes in functional connectivity between patients and HC over time. Simple main effect indicated that at T2, the strength of functional connectivity in the SMA‐S1 and SMA‐sensorimotor cerebellum of CD patients was significantly lower than that observed in HCs (*p* = 0.042, *p* = 0.032, respectively). Conversely, at T3, except for M1‐S1 and M1‐sensorimotor cerebellum, all functional connectivity strengths in CD patients were significantly higher than those in HCs. Notably, the functional connection involving the S1‐sensorimotor cerebellum exhibited the most substantial difference (*F* = 15.838, *p* < 0.001), indicating a notable difference in neural interaction and communication between these crucial motor areas in patients compared with HCs over time.

Overall, there was no significant difference in functional connectivity between CD patients and HCs during the preparation stage for executing ST. However, during the maintenance period of ST, the neural interactions and communication among key motor regions in CD patients are significantly enhanced compared with HCs. Conversely, in the initial stages of ST, the strength of functional connectivity between the SMA and other regions in CD patients is significantly lower than that observed in HC, suggesting a diminished initial neural coordination and communication within the motor network of patients, highlighting a potential impairment or delayed activation in integrating sensory and motor information at the outset of the task.

Additionally, to objectively quantify the severity of the patients' condition, we conducted a linear regression analysis between the disease severity, as assessed by the scale, and the strength of the patients' functional connectivity at rest (baseline). The results of the analysis unveil a significant inverse relationship between the functional connectivity strength of the SMA‐M1 and SMA‐S1 in patients and the severity of their condition. Specifically, during the resting period, it was observed that lower functional connectivity strength of the SMA‐M1 and SMA‐S1 is correlated with higher severity of the illness. This observation suggests that the integrity of the neural connection between these pivotal motor and sensory regions plays a crucial role in the manifestation of disease symptoms, with decreased connection strength potentially reflecting more severe neurological impairment. This finding could offer valuable insights for designing targeted interventions aimed at enhancing neural communication within these regions to mitigate disease severity.

Here, we also explored differences of the source power and functional connections in alpha and beta bands between two groups (Tables S3 and S4).

## Discussion

**Figure 5:**
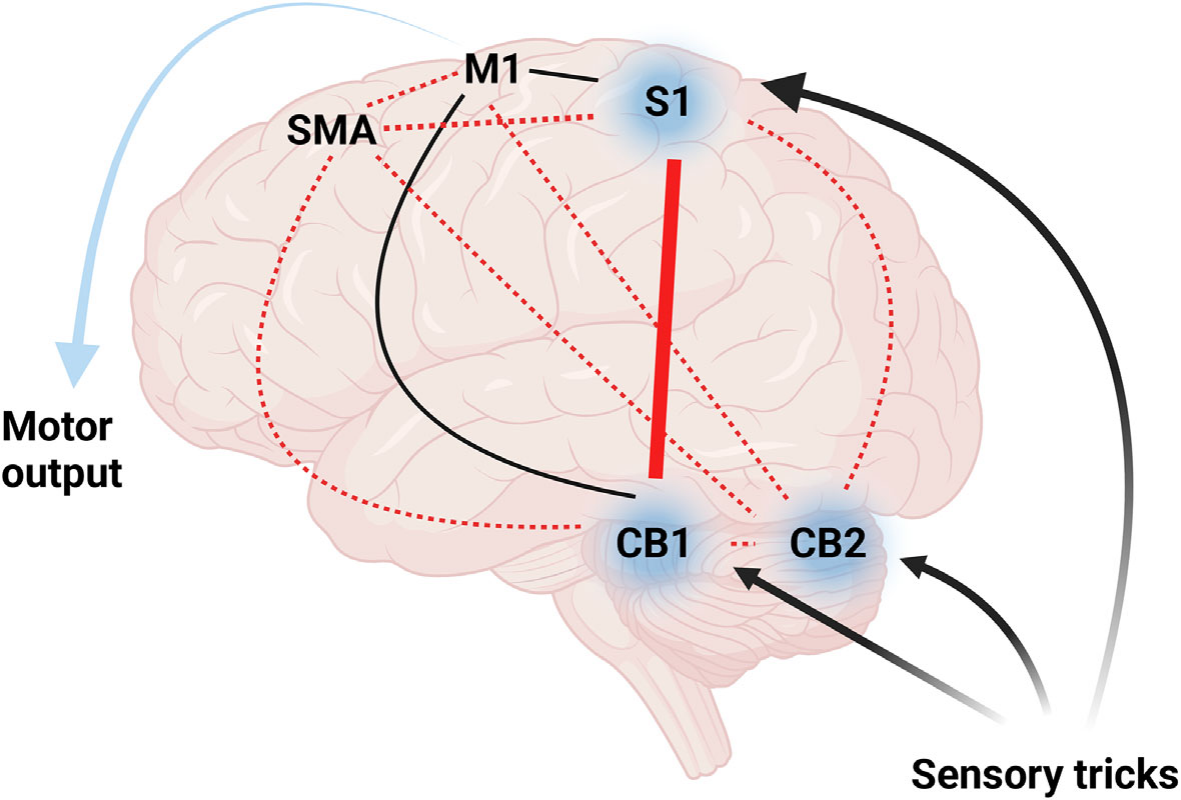
Hypothetical diagram of the pathophysiological mechanisms of ST. When ST being implemented, the information of sensory input is transmitted to S1 and the cerebellar functional regions (blue background), which results in a decrease of gamma band power in these areas and a significant increase of the connectivity in S1‐CB1, and then, the dystonic posture is relieved. The red solid line represents very significant strengthening of connectivity; the red broken lines represent strengthening of connectivity; and the black solid lines represent no strengthening of connectivity. CB1, sensorimotor cerebellum; CB2, cognitive cerebellum; M1, primary motor cortex; S1, primary sensory cortex; SMA, supplementary motor area.

We conducted an EEG source intensity study involving 15 CD patients with ST and 15 HCs. Our aim was to explore the cerebral‐cerebellar‐cortical excitability and connectivity of ST effects. The main findings of this study are as follows: (1) The application of ST led to a reduction of low gamma band activity in S1, sensorimotor cerebellum, and cognitive cerebellum cortices. This reduction could not be solely attributed to motor processing. (2) Compared with HCs, CD patients exhibited consistent strengthening of connectivity within the sensorimotor network during the maintenance period, particularly in the S1‐sensorimotor cerebellum connection. (3) The activation pattern of the sensorimotor cerebellum and cognitive cerebellum in CD patients differed from that of HCs during action maintenance, suggesting that cerebellar functional regions might play an essential role in the persistence of ST effects. (Fig. 5).

### ST changes cortical activity

We can confirm that the altered cortical activation we observed cannot be solely attributed to changes in cortical recruitment due to motor processing. During the application of ST, the source‐based spectral power of CD patients decreased significantly over S1, sensorimotor cerebellum, and cognitive cerebellum areas, remaining lower than that of HCs during the maintenance period. In contrast, the HC group, when mimicking ST, exhibited changes in spectral power over these areas, returning close to levels observed during the preparation period. This finding is consistent with previous research on sensorimotor integration disorder in dystonia.^8^, ^22^ The changes induced by ST in the activity of S1 and cerebellar areas, rather than the motor cortex, underscore the importance of sensory input and integration in the mechanism of ST. Previous EEG studies have also highlighted the role of sensorimotor areas in the ST procedure, emphasizing the contribution of sensory components to the ST effects.^12^

We mainly observed the ST effects in the low gamma band based on TF analysis, in line with the functions of neural oscillations as revealed by previous studies. Gamma oscillations are closely linked to the selective transmission of sensory information across distributed neurocircuits, modulating neurocircuit function, behavior, and memory.^23^ They also play a role in motor performance, as evidenced in individuals with Parkinson's disease^24^, ^25^ and isolated dystonia.^26^, ^27^, ^28^, ^29^ Animal experiments further support the involvement of gamma oscillations in sensorimotor integration. For instance, optogenetically induced 40 Hz gamma oscillations in the barrel cortex of mice temporally sharpen the evoked spiking response of sensory‐whisker deflections.^30^ In our study, the role of gamma band within the sensorimotor integration mechanism was emphasized in the ST effects, which differs from some other similar studies.^11^, ^12^ However, we acknowledge that the role of other frequency bands cannot be ruled out.

### ST facilitates the connections between sensorimotor cortices

Previous studies on brain networks have supported the concept that CD is a disorder of the sensorimotor circuit, with a focus on the pallidum, S1, SMA, precuneus, occipital lobe, and cerebellum as important junctions.^31^, ^32^, ^33^ Signs of hyper‐ and hypo‐connectivity have been observed in bilateral regions of the sensorimotor network, suggesting sensorimotor integration dysfunction underlying the pathophysiology of CD.^34^ However, the changes in cortical connections related to ST remain unknown.

Our network analysis revealed that ST could facilitate cortical connections within the sensorimotor circuit. Compared with HCs, CD patients exhibited consistent strengthened connectivity within the sensorimotor network during the maintenance period, particularly in the S1‐sensorimotor cerebellum connection. We also explored the dynamic changes in functional connectivity in CD patients compared with HCs, revealing a temporary decrease in functional connectivity of SMA‐sensorimotor cerebellum and SMA‐S1, followed increases in connectivity between overall ROIs except M1‐S1 and M1‐sensorimotor cerebellum. These findings align partly with two similar fMRI studies indirectly investigating network alterations in ST. Sarasso *et al*.^9^ observed increased activation in overall sensorimotor regions during sensory trick imagination in CD patients, particularly in the cerebellum, which corresponds to our results. Another study demonstrated increased SMA connectivity in brain regions involved in sensorimotor integration during sensory trick performance and imagination.^35^

### Cerebellum involves in the ST effect maintenance

Our results highlight the significance of S1 in CD pathophysiology and the effect of ST, acknowledged.^4^, ^8^, ^36^ Another crucial finding of our study was the alteration in cerebellar activation patterns observed in CD patients during maneuver maintenance, aligning with our earlier hypotheses regarding the involvement of the cerebellum in the ST effects.

The cerebellum plays a pivotal role in controlling, coordinating, and planning voluntary movements, thereby modulating sensorimotor integration. Numerous clinical studies have emphasized the cerebellum's importance in dystonia pathophysiology.^37^ Animal experiments have demonstrated the connection between the cerebellum and the basal ganglia via the cerebellar‐basal ganglia‐thalamo‐cortical loop, mediated by the neurotransmitter gamma‐aminobutyric acid (GABA) from GABAergic Purkinje cells in the cerebellar cortex.^13^, ^14^ The association between cerebellum and CD mechanism is increasingly recognized. Prudente *et al*.^38^ observed patchy loss of Purkinje cells in the cerebellum's postmortem brain tissue from individuals with CD, potentially leading to iron overload and axonal damage.^39^

Furthermore, as previously mentioned, the cerebellar structure distinguishes between the anterior lobe and the posterior lobe,^15^ suggesting different roles for various cerebellar regions in CD. Our findings support this notion and further investigate their contribution to the ST effect. To be specific, ST induced a reduction in low gamma band spectral power and maintained it at a low level over sensorimotor and cognitive cerebellar areas, while strengthening the S1‐sensorimotor cerebellum connection. To our knowledge, we are the first to apply this approach to study the cortical correlates of the ST effect in CD patients.

### Study limitations

The interpretation of the results from our study should be approached with certain limitations in mind. Firstly, it is important to note that the positions of the ST may vary among individuals with CD, encompassing differences in unilateral maneuver execution, responsiveness time following prompts, and subjectivity in the observed effects of ST. To mitigate potential confounding factors, we implemented several methodological precautions:

1. Before including CD patients in the final analysis, we assessed the improvement rate of ST and EMG to ensure objective effectiveness of the ST. Patients displaying obvious tremor or jerk were excluded to minimize bias related to tremors and reduce EEG interference, albeit resulting in a reduction of the sample size.
2. Movement onset and execution were self‐paced to emulate real‐world features and effects associated with ST. To maintain consistency, we utilized signals from the bicep brachii muscle EMG as markers for time division.
3. We did not impose limitations based on hemispheric dominance to replicate real‐world ST‐related features. Patients were instructed to perform the most effective ST, aiming to make cortical activity in EEG data more significant and reliable. To mitigate potential bias, we standardized the ipsilateral hemisphere of all subjects to the left brain by referencing the medial interhemispheric fissure line. Additionally, HCs were all right‐handed.

In combination, our findings may underestimate the true changes in cortical activation induced by ST.

Another limitation to consider is the spatial resolution of EEG, which, despite its excellent temporal resolution, remains constrained. Additionally, it is virtually impossible to explore the role of deeper, subcortical structures such as the basal ganglia. The cerebellum is also challenging to assess using EEG. Our current understanding of cerebellar electrophysiological mechanisms primarily stems from direct recordings in animals, while investigations into cerebellar function in humans have predominantly relied on lesion studies, as well as haemodynamic and metabolic imaging studies. Although these approaches offer fundamental insights into cerebellar‐cortical pathways, they are limited in temporal and spectral resolution.

Some perspectives suggest that non‐invasive detection of cerebellar activity with MEG and EEG is feasible and can be enhanced with appropriate methods, particularly using connectivity analysis in source space.^40^ In this study, we optimized the detection of cerebellar activity with EEG by employing source‐based EEG and functional connectivity. Theoretically, EEG is suitable for researching ST mechanisms, and the outcomes regarding the cerebellum in our study are relatively reliable. However, the primary advantage of using EEG over neuroimaging techniques with higher spatial resolution is the ability to measure cerebral‐cerebellar‐cortical activity during ST rather than relying on imagined tasks in a scanner. In future studies, employing more high‐density EEG (e.g., with more than 128 channels) could provide additional information.

Other limitations of our study included the observation of only low gamma band activity as previously discussed, and none age‐and sex‐matching. Additionally, the choice of ROIs was arbitrary based on previous studies and observed unilaterally, potentially overlooking interhemispheric communications underlying ST mechanisms. This aspect could be an intriguing subject for future research.

## Conclusions

This study offers insights into the differential changes in cerebral‐cerebellar‐cortical activity and functional networks regulated by gamma oscillations between CD patients and HCs during ST or ST mimicking, as observed from the EEG source level. It provides evidence for the involvement of the cerebellum in the ST effects.

Specifically, our findings support the engagement of various cerebellar functional regions and illustrate their potential patterns associated with ST. These observations may offer new perspectives for future treatments for dystonia.

## Author Contributions

NQC, YRL, and NW contributed to the conception and design of the study; WXS, RKC, and BLC contributed to the acquisition and analysis of data; NQC, WXS, and RKC contributed to drafting the text or preparing the figures.

## Conflict of Interests

Nothing to report.

## Supporting information


Table S1.

Table S2.

Table S3.

Table S4.

## Data Availability

Data are available upon reasonable request to the corresponding authors.
