# Supplementary material for: Cerebral‐Cerebellar Cortical Activity and Connectivity Underlying Sensory Trick in Cervical Dystonia
Source: Ann Clin Transl Neurol. 2024 Aug 16;11(10):2633–44. doi: 10.1002/acn3.52177 (PMC11514925; doi:10.1002/acn3.52177)
Supplement: Supplementary file 1 — Table S1. [file ACN3-11-2633-s003.docx]

Supplementary Table 1. Repeated Measure ANOVA Analyses for Source Power on the Gamma Band

| Factor | Level | df | F | p value |
| --- | --- | --- | --- | --- |
| Mixed ANOVA |  |  |  |  |
| Time |  | 2;27 | 0.439 | 0.648 |
| Group |  | 1;28 | 2.290 | 0.142 |
| Region |  | 4;25 | 1.897 | 0.171 |
| Time*Group |  | 2;27 | 2.414 | 0.109 |
| Time*Region |  | 8;21 | 5.746 | 0.005 |
| Group*Region |  | 4;25 | 8.220 | 0.003 |
| Time*Group*Region |  | 8;21 | 3.738 | 0.030 |
| Post-hoc Analysis |  |  |  |  |
| Group*Region | T1 | 4;25 | 0.410 | 0.598 |
| Group*Region | T2 | 4;25 | 6.554 | 0.010 |
| Group*Region | T3 | 4;25 | 3.782 | 0.031 |
| Simple Main Effect on T2 | | | | |
| Group | T2*M1 | 1;28 | 0.290 | 0.594 |
| Group | T2*SMA | 1;28 | 3.088 | 0.090 |
| Group | T2*S1 | 1;28 | 0.045 | 0.833 |
| Group | T2*CB1 | 1;28 | 3.995 | 0.056 |
| Group | T2*CB2 | 1;28 | 4.275 | 0.048 |
| Simple Main Effect on T3 | | | | |
| Group | T3*M1 | 1;28 | 0.450 | 0.508 |
| Group | T3*SMA | 1;28 | 0.139 | 0.712 |
| Group | T3*S1 | 1;28 | 4.366 | 0.046 |
| Group | T3*CB1 | 1;28 | 16.857 | <0.001 |
| Group | T3*CB2 | 1;28 | 19.692 | <0.001 |

M1 = primary motor cortex; SMA = supplementary motor area; S1 = primary sensory cortex; CB1 = sensorimotor cerebellum; CB2 = cognitive cerebellum.
