# Supplementary material for: Cerebral‐Cerebellar Cortical Activity and Connectivity Underlying Sensory Trick in Cervical Dystonia
Source: Ann Clin Transl Neurol. 2024 Aug 16;11(10):2633–44. doi: 10.1002/acn3.52177 (PMC11514925; doi:10.1002/acn3.52177)
Supplement: Supplementary file 2 — Table S2. [file ACN3-11-2633-s004.docx]

Supplementary Table 2. Repeated Measure ANOVA Analyses for Functional Connectivity on the Gamma Band

| Factor | Level | df | F | p value |
| --- | --- | --- | --- | --- |
| Mixed ANOVA |  |  |  |  |
| Time |  | 2;27 | 2.152 | 0.128 |
| Group |  | 1;28 | 4.381 | 0.046 |
| Region |  | 9;20 | 31.486 | <0.001 |
| Time*Group |  | 2;27 | 8.101 | 0.001 |
| Time*Region |  | 18;11 | 0.585 | 0.748 |
| Group*Region |  | 9;20 | 1.186 | 0.321 |
| Time*Group*Region |  | 18;11 | 1.388 | 0.220 |
| Simple Main Effect on T1 | | | | |
| Group | T1*M1-SMA | 1;28 | 0.029 | 0.866 |
| Group | T1*M1-S1 | 1;28 | 0.152 | 0.699 |
| Group | T1*M1-CB1 | 1;28 | 0.094 | 0.762 |
| Group | T1*M1-CB2 | 1;28 | 0.298 | 0.590 |
| Group | T1*SMA-S1 | 1;28 | 0.001 | 0.988 |
| Group | T1*SMA-CB1 | 1;28 | 0.235 | 0.632 |
| Group | T1*SMA-CB2 | 1;28 | 0.687 | 0.414 |
| Group | T1*S1-CB1 | 1;28 | 0.001 | 0.997 |
| Group | T1*S1-CB2 | 1;28 | 0.161 | 0.691 |
| Group | T1*CB1-CB2 | 1;28 | 0.032 | 0.860 |
| Simple Main Effect on T2 | | | | |
| Group | T2*M1-SMA | 1;28 | 0.766 | 0.389 |
| Group | T2*M1-S1 | 1;28 | 1.886 | 0.181 |
| Group | T2*M1-CB1 | 1;28 | 0.351 | 0.558 |
| Group | T2*M1-CB2 | 1;28 | 0.901 | 0.351 |
| Group | T2*SMA-S1 | 1;28 | 4.580 | 0.042 |
| Group | T2*SMA-CB1 | 1;28 | 3.959 | 0.057 |
| Group | T2*SMA-CB2 | 1;28 | 5.082 | 0.032 |
| Group | T2*S1-CB1 | 1;28 | 0.154 | 0698 |
| Group | T2*S1-CB2 | 1;28 | 0.001 | 0.977 |
| Group | T2*CB1-CB2 | 1;28 | 1.158 | 0.291 |
| Simple Main Effect on T3 | | | | |
| Group | T3*M1-SMA | 1;28 | 10.691 | 0.003 |
| Group | T3*M1-S1 | 1;28 | 0.938 | 0.341 |
| Group | T3*M1-CB1 | 1;28 | 3.921 | 0.058 |
| Group | T3*M1-CB2 | 1;28 | 11.055 | 0.003 |
| Group | T3*SMA-S1 | 1;28 | 8.414 | 0.007 |
| Group | T3*SMA-CB1 | 1;28 | 4.883 | 0.036 |
| Group | T3*SMA-CB2 | 1;28 | 10.371 | 0.003 |
| Group | T3*S1-CB1 | 1;28 | 4.823 | 0.037 |
| Group | T3*S1-CB2 | 1;28 | 15.838 | <0.001 |
| Group | T3*CB1-CB2 | 1;28 | 6.817 | 0.015 |

M1= primary motor cortex; SMA = supplementary motor area; S1 = primary sensory cortex; CB1 = sensorimotor cerebellum; CB2 = cognitive cerebellum.
