# Supplementary material for: Cerebral‐Cerebellar Cortical Activity and Connectivity Underlying Sensory Trick in Cervical Dystonia
Source: Ann Clin Transl Neurol. 2024 Aug 16;11(10):2633–44. doi: 10.1002/acn3.52177 (PMC11514925; doi:10.1002/acn3.52177)
Supplement: Supplementary file 3 — Table S3. [file ACN3-11-2633-s002.docx]

**Supplementary Table 3. Repeated Measure ANOVA Analyses for Source Power on the Alpha and Beta Bands**

| Factor | Level | df | F | p |
| --- | --- | --- | --- | --- |
| **Alpha** |  |  |  |  |
| Time |  | 2;27 | 0.680 | 0.511 |
| Group |  | 1;28 | 2.492 | 0.126 |
| Region |  | 4;25 | 9.652 | 0.002 |
| Time*Group |  | 2;27 | 2.218 | 0.122 |
| Time*Region |  | 8;21 | 4.183 | 0.015 |
| Group*Region |  | 4;25 | 0.355 | 0.617 |
| Time*Group*Region |  | 8;21 | 0.594 | 0.581^#^ |
| **Beta** |  |  |  |  |
| Time |  | 2;27 | 5.705 | 0.006 |
| Group |  | 1;28 | 0.857 | 0.363 |
| Region |  | 4;25 | 15.107 | <0.001 |
| Time*Group |  | 2;27 | 0.518 | 0.592 |
| Time*Region |  | 8;21 | 4.933 | 0.006 |
| Group*Region |  | 4;25 | 2.723 | 0.096 |
| Time*Group*Region |  | 8;21 | 1.666 | 0.190^#^ |

#: The results illustrated that there is no significant difference in source power between CD patients and HCs within the alpha and beta frequency bands.
