# Supplementary material for: Cerebral‐Cerebellar Cortical Activity and Connectivity Underlying Sensory Trick in Cervical Dystonia
Source: Ann Clin Transl Neurol. 2024 Aug 16;11(10):2633–44. doi: 10.1002/acn3.52177 (PMC11514925; doi:10.1002/acn3.52177)
Supplement: Supplementary file 4 — Table S4. [file ACN3-11-2633-s001.docx]

**Supplementary Table 4. Repeated measure ANOVA for functional connectivity on the alpha and Beta band**

| Factor | Level | df | F | p |
| --- | --- | --- | --- | --- |
| **Alpha** |  |  |  |  |
| Time |  | 2;27 | 0.007 | 0.993 |
| Group |  | 1;28 | 1.057 | 0.314 |
| Region |  | 9;20 | 18.658 | <0.001 |
| Time*Group |  | 2;27 | 0.825 | 0.444 |
| Time*Region |  | 18;11 | 0.802 | 0.541 |
| Group*Region |  | 9;20 | 1.622 | 0.200 |
| Time*Group*Region |  | 18;11 | 0.995 | 0.420 |
| **Beta** |  |  |  |  |
| Time |  | 2;27 | 0.114 | 0.892 |
| Group |  | 1;28 | 0.170 | 0.683 |
| Region |  | 9;20 | 34.515 | <0.001 |
| Time*Group |  | 2;27 | 0.087 | 0.908 |
| Time*Region |  | 18;11 | 1.365 | 0.229 |
| Group*Region |  | 9;20 | 0.806 | 0.515 |
| Time*Group*Region |  | 18;11 | 2.511 | 0.022^#^ |
| Simple Main Effect on T1 |  |  |  |  |
| Group | T1*M1-SMA | 1;28 | 0.052 | 0.822 |
| Group | T1*M1-S1 | 1;28 | 1.529 | 0.227 |
| Group | T1*M1-CB1 | 1;28 | 0.078 | 0.782 |
| Group | T1*M1-CB2 | 1;28 | 0.186 | 0.670 |
| Group | T1*SMA-S1 | 1;28 | 0.167 | 0.686 |
| Group | T1*SMA-CB1 | 1;28 | 0.086 | 0.772 |
| Group | T1*SMA-CB2 | 1;28 | 0.197 | 0.661 |
| Group | T1*S1-CB1 | 1;28 | 0.178 | 0.677 |
| Group | T1*S1-CB2 | 1;28 | 0.092 | 0.764 |
| Group | T1*CB1-CB2 | 1;28 | 0.189 | 0.668 |
| Simple Main Effect on T2 |  |  |  |  |
| Group | T2*M1-SMA | 1;28 | 8.655 | 0.007^#^ |
| Group | T2*M1-S1 | 1;28 | 1.839 | 0.186 |
| Group | T2*M1-CB1 | 1;28 | 0.983 | 0.330 |
| Group | T2*M1-CB2 | 1;28 | 1.788 | 0.192 |
| Group | T2*SMA-S1 | 1;28 | 4.369 | 0.046^#^ |
| Group | T2*SMA-CB1 | 1;28 | 0.050 | 0.826 |
| Group | T2*SMA-CB2 | 1;28 | 0.081 | 0.779 |
| Group | T2*S1-CB1 | 1;28 | 1.972 | 0.172 |
| Group | T2*S1-CB2 | 1;28 | 2.592 | 0.119 |
| Group | T2*CB1-CB2 | 1;28 | 2.607 | 0.118 |
| Simple Main Effect on T3 |  |  |  |  |
| Group | T3*M1-SMA | 1;28 | 1.192 | 0.285 |
| Group | T3*M1-S1 | 1;28 | <0.001 | 0.990 |
| Group | T3*M1-CB1 | 1;28 | 0.960 | 0.336 |
| Group | T3*M1-CB2 | 1;28 | 1.486 | 0.233 |
| Group | T3*SMA-S1 | 1;28 | 2.923 | 0.099 |
| Group | T3*SMA-CB1 | 1;28 | 0.652 | 0.427 |
| Group | T3*SMA-CB2 | 1;28 | 2.682 | 0.113 |
| Group | T3*S1-CB1 | 1;28 | 0.985 | 0.330 |
| Group | T3*S1-CB2 | 1;28 | 1.819 | 0.189 |
| Group | T3*CB1-CB2 | 1;28 | 1.379 | 0.250 |

#: The results illustrated that there are significant differences in functional connectivity between CD patients and HCs in the M1-SMA and SMA-S1 pathways.

M1= primary motor cortex; SMA = supplementary motor area; S1= primary sensory cortex; CB1= sensorimotor cerebellum; CB2= cognitive cerebellum.
